# GesturalOrigins: A bottom-up framework for establishing systematic gesture data across ape species

**DOI:** 10.3758/s13428-023-02082-9

**Published:** 2023-03-15

**Authors:** Charlotte Grund, Gal Badihi, Kirsty E. Graham, Alexandra Safryghin, Catherine Hobaiter

**Affiliations:** School of Psychology and Neuroscience, University of St Andrews, Fife, Scotland, KY16 9JP UK

**Keywords:** Video coding, Gesture action phases, GesturalOrigins, Visual communication, Language evolution

## Abstract

Current methodologies present significant hurdles to understanding patterns in the gestural communication of individuals, populations, and species. To address this issue, we present a bottom-up data collection framework for the study of gesture: GesturalOrigins. By “bottom-up”, we mean that we minimise *a priori* structural choices, allowing researchers to define larger concepts (such as ‘gesture types’, ‘response latencies’, or ‘gesture sequences’) flexibly once coding is complete. Data can easily be re-organised to provide replication of, and comparison with, a wide range of datasets in published and planned analyses. We present packages, templates, and instructions for the complete data collection and coding process. We illustrate the flexibility that our methodological tool offers with worked examples of (great ape) gestural communication, demonstrating differences in the duration of action phases across distinct gesture action types and showing how species variation in the latency to respond to gestural requests may be revealed or masked by methodological choices. While GesturalOrigins is built from an ape-centred perspective, the basic framework can be adapted across a range of species and potentially to other communication systems. By making our gesture coding methods transparent and open access, we hope to enable a more direct comparison of findings across research groups, improve collaborations, and advance the field to tackle some of the long-standing questions in comparative gesture research.

## Introduction: Establishing systematic data in comparative great ape gesture research

Gestural signals are employed across a diverse range of species (e.g., baboons: Molesti et al., 2020; mangabeys: Schel et al., 2022; macaques: Gupta & Sinha, 2019; ravens: Pika & Bugnyar, 2011; grouper fish: Vail et al., 2013), but most gesture research has focussed on great apes (e.g., chimpanzees: Tomasello et al., 1985; Liebal et al., 2006; Hobaiter & Byrne, 2011a; bonobos: Pika et al., 2005; Graham et al., 2017; gorillas: Pika et al., 2003; Genty et al., 2009; orang-utans: Liebal et al., 2006; Cartmill & Byrne, 2007). Despite working on the same or closely related species, a diverse range of methods to investigate ape gesture has emerged over the last four decades. Variation in approach, including different methods, research settings, and species, is essential for the robust exploration of behaviour (Rodrigues et al., 2021), but the diversity of approaches to studying ape gesture has raised hurdles for cross-study comparability, and recent reviews have highlighted the need for more methodological transparency (e.g., Scott & Pika, 2012; Fröhlich & Hobaiter, 2018; Bourjade et al., 2020; Rodrigues et al., 2021). Our ability to tackle comparative questions is not dependent on the adoption of a universal approach; but it is dependent on methods that are sufficiently transparent and detailed to allow the direct comparison of like-with-like data. Given the scale of the question and the nature of studying long-lived, behaviourally flexible, and highly social individuals, the most straightforward and effective way to investigate gestural behaviour in apes and similar species is to collaborate and compare findings across studies.

This challenge is not unique to ape gesture research: recent work on the ‘replication crisis’ (Open Science Collaboration, 2015; Baker, 2016) highlights issues in the reliability of findings across behavioural research (Webster & Rutz, 2020; Rutz & Webster, 2021). While it may sometimes be impossible or inappropriate to exactly replicate data (Farrar et al., 2021), variability becomes an issue when the sources of variation are opaque – without transparent methods, we are unable to discern whether differences in results come, for example, from sampling biases or methodological misconceptions (Schweinfurth & Call, 2021). One means to address this challenge is through improved transparency in describing data – for example, the STRANGE framework clarifies the nature of sampling biases in animal research (Webster & Rutz 2020) by taking a ‘design, declare, and discuss’ approach. In tandem, bottom-up methodological frameworks allow researchers to construct and reconstruct datasets in different ways, providing both individual flexibility and the opportunity for like-with-like comparison (where the raw data are made available; Eaton et al., 2018).

We aim to contribute to an increase in methodological robustness and transparency in gesture research by presenting a bottom-up data collection framework: GesturalOrigins. We aim to provide enough detail for this framework to be easily adopted by other researchers. We integrate and build upon decades of methodological advances in gesture research and present packages, templates, and instructions for the complete data collection process: from collecting interactional video data; to coding gestural communication events in ELAN, an accessible open-source software (ELAN, 2022); to exporting coded data and transforming it for storage in a suitable database, e.g., Filemaker. We use practical examples of chimpanzee and gorilla gestural communication to demonstrate the adaptability of our framework, providing a video tutorial and protocols for the data curation procedure (collection, coding, and export) that also outline the theoretical decision-making process involved. As primatologists, we take a primate-centred perspective, but the basic framework may potentially be adapted across a range of species (e.g., African savannah elephants, see the Wild Minds lab homepage https://www.wildminds.ac.uk/ for ongoing projects).

## The GesturalOrigins coding framework

### Basic considerations when measuring gestural behaviour

Observational studies of animal behaviour provide rich, nuanced descriptions of how individuals, groups, and species interact with their physical and social environments (Altmann, 1974). However, direct in-person observation is vulnerable to a range of biases in attention and perception (Kaufman & Rosenthal, 2009). Gesture data include variables that are especially prone to direct observation error as they rely on timings in the realm of seconds (e.g., in the case of ‘response waiting’, Table 1 next section); on subtle differences in behaviour (e.g., changes in the direction of eye gaze (‘visual attention’), Table 1 next section) or action movements (e.g., to determine the difference between a ‘swing’ and a ‘fling’ in a social interaction where a lot is happening at once); and often involve the collection of data points from multiple individuals that may overlap in time. Especially in naturalistic conditions where observation is further impeded by visually dense environments and variable lighting, gestural behaviour cannot be collected reliably during live focal sampling. Video-based coding, considered a gold standard in observational research (Gilmore & Adolph, 2017), not only provides precise time measurements and identification of subtle, as well as obvious actions, but also allows researchers to revisit interactions, improve training of new coders, and facilitates the accurate completion of important intra-coder and inter-coder reliability tests (Burghardt et al. 2012). In-video annotation, where coded data are directly linked to the video material, further increases accuracy, and eases the burden of revisiting data to assess reliability or generate comparisons. There are several in-video behavioural coding programs available, for example BORIS (Friard & Gamba, 2016), Observer XT (Noldus et al., 2000), Solomon Coder (Péter, 2011) or ELAN (ELAN, 2022), to name a few. Inspired by previous video-based communication research (e.g., Heesen et al., 2020) we implemented our coding scheme in the open-source linguistic video annotation software ELAN (ELAN, 2022). We describe step‐by‐step how to code with the GesturalOrigins coding scheme in our coding protocol (GOv1.0_Protocol), accessible through the electronic supplementary material (ESM) of this manuscript on GitHub (https://github.com/CharlotteGrund/Gestural_Origins_Coding-methods_paper, along with all other files referred to as ESM throughout the manuscript). Besides detailed descriptions of all our variables, the protocol includes sections on how to export the coded data from ELAN and offers R packages that convert the export file into a ‘clean’ data file for further analysis.

### What counts as gestural behaviour? Behavioural indicators of intention

In line with a large body of non-human animal gesture research (Graham et al., 2017; Gupta & Sinha, 2019; Hobaiter & Byrne, 2011a; Liebal et al., 2006; Molesti et al., 2020; Pika & Bugnyar, 2011; Schel et al., 2022; Tomasello et al., 1985; Vail et al., 2013), we define a gesture as a signal produced with the body, which is a “mechanically ineffective physical movement” (Hobaiter & Byrne, 2011a) of a body part/parts or the body as a whole, used intentionally to achieve a specific social goal (Bard, 1992; Leavens & Hopkins 1998; Pika et al., 2003; Pollick & de Waal, 2007; Tomasello et al., 1985; Hobaiter & Byrne, 2011a). A gesture is produced voluntarily by the signalling individual (i.e., it is not a reflexive or automatic reaction to some external or internal cue) and is aimed at eliciting a particular behavioural response from a specific individual (the recipient). The communication is successful when the recipient changes their behaviour in a way that represents a plausible goal from the signaller’s perspective (the apparently satisfactory outcome (ASO), usually a form of social interaction) and the signaller stops signalling (indicating that she was apparently satisfied by the change in behaviour). A gesture may involve mechanical manipulation of the recipient by the signaller (as e.g., in a ‘push’ gesture – see ESM: GOv1.0_Gesture_action_definitions.xlsx for a definition of gesture actions) but never to an extent that fulfils the goal itself (the action is *mechanically ineffective*: a ‘push’ may be used to signal to the recipient to move his body in a certain direction, but the force used should not be effective in moving the recipient’s body to the desired location).

Non-human animal gesture researchers exapted intentionality criteria for non-human communication from research on intentionality in human children’s pre-verbal communication and their shift from perlocutionary to illocutionary communicative acts (Bates et al. 1975, summarised in Table 1 below). While the presence of individual markers such as these can be explained in other ways, together their combined and regular use provide confidence that the signallers’ communication is best understood as intentional.

**Table 1:** Definitions of behavioural proxies used to identify intentional gestural signalling

Term	Definition
Audience checking	the signaller checks the recipient’s state of visual attention before the production of the signal and adjusts her signalling accordingly (e.g., using visual-only signals when the recipient is looking and audible or tactile ones when he is not, increasing the changes of a signal being perceived and showing so-called “sensitivity to the attentional state”)
Response waiting	the signaller pauses and waits for the recipient to respond to his request (behavioural cue here is a pause in gesturing and the visual monitoring of the recipient)
Goal persistence	the signaller continues to signal when the recipient does not respond and either persists and/or elaborates with more gesturing until the goal is met
Mechanical ineffectiveness	the signaller’s gesture may (mechanically) manipulate the recipient but never to an extent that fulfils the goal itself (the gesture action is *mechanically ineffective*: a ‘push’ may be used to signal to the recipient to move his body in a certain direction, but the force used should not be effective in moving the recipient’s body to the desired location)

In our coding routine, we pre-screen all collected video clips for potential gestural behaviour (typically where two (or more) individuals are within 5 m of each other and show some form of social engagement) and apply the intentionality criteria described in Table 1 to triage actual instances of gesture use for detailed coding. Although we focus our description here on clearly intentional gestural signals in dyadic interactions, the GesturalOrigins coding scheme can equally be applied to examples of gesture use that appear to target multiple individuals or where the criteria for intentional use are not clear. This openness means there is no need to decide *a priori* to only code intentional goal-directed instances – these can instead be subsetted from the data afterwards depending on which criteria are then applied. Coding in this way substantially increases the coding effort needed to detect gestures but may be of interest in studies wishing to discriminate how intentional gestural actions vary from other physical signals or when investigating varying degrees of intentionality in signal production (e.g., during ontogeny). Moreover, this also provides the option of exploring the usefulness of these markers for establishing intentional use across gesture types (for example, there is an established bias towards visual signals (Dafreville et al., 2021)). Improving markers for intentionality for the various modalities in which gestures and other signal types occur is a scientific challenge (e.g., Townsend et al., 2017; Fröhlich & Hobaiter, 2018), and we hope that bottom-up coding schemes will prove valuable tools for data-driven approaches to address some of these challenges.

### What unit constitutes a gesture? Describing gesture instances (physical form)

While there is widespread agreement on how to define gestural behaviour and apply its behavioural markers, there remains more substantial variation in the methodologies used to identify and describe the physical forms of the gestural signals (Rodrigues et al., 2021; Hobaiter & Byrne, 2017). Studies vary in what actions or body parts they consider; some include whole-body postures (e.g., Hobaiter & Byrne, 2011a; Molesti et al., 2020) and/or ‘facial gestures’ (e.g., Cartmill & Byrne, 2010; Molesti et al., 2020), while others are restricted only to movements of the hands and arms (e.g., Pollick & de Waal, 2007; Roberts et al., 2012). In a recent systematic literature review of human and non-human gesture, Rodrigues et al. (2021) found that in 163 studies 54% of researchers only included manual movements, 34% included both manual and nonmanual, and the remainder either did not specify the body part or included only nonmanual gestures. Approaches to gesture type construction also deviate in how fine-grained they split actions based on body parts involved (e.g., some studies classify actions produced with one or both hands as different ‘gesture types’, e.g., Genty et al., 2009) or in how far they discriminate functional aspects of a gesture that are independent of its morphological appearance (e.g., ‘present grooming’ and ‘present sexual’ may involve the exact same movement in different behavioural contexts; e.g., Hobaiter & Byrne, 2011a). Thus, despite common elements typically used to describe a gesture ‘type’: actions, body parts, the involvement of objects, the use of repetition (Hobaiter & Byrne, 2017), researchers often recombine these in non-systematic ways across studies.

Together these methodological inconsistencies can result in divergent outcomes between studies with respect to gesture types, repertoire sizes, and number of gesture instances observed, even within an identical set of ape behavioural video. In some cases, comparisons may still be feasible by lumping up (for example, combining all the unimanual and bimanual actions) but doing so may lose important resolution. For example, excluding gestures conducted with the body or body parts other than the limbs would reduce the chimpanzee repertoire described by Hobaiter et al. (2011a) by ~26%. Importantly, there is no ‘right’ way to analyse gesture, but where each coding scheme is adapted to the particular questions of interest, the raw coded data are biased in ways that make comparisons between studies challenging. Potential for comparison across studies is particularly limited where definitions are not transparent, or the construction of gesture types does not follow consistent rules. Our approach addresses this challenge by coding gesture units from the bottom-up, allowing researchers to describe gesture ‘types’ at different levels and with data-driven probabilistic approaches post-coding.

**Fig. 1:**
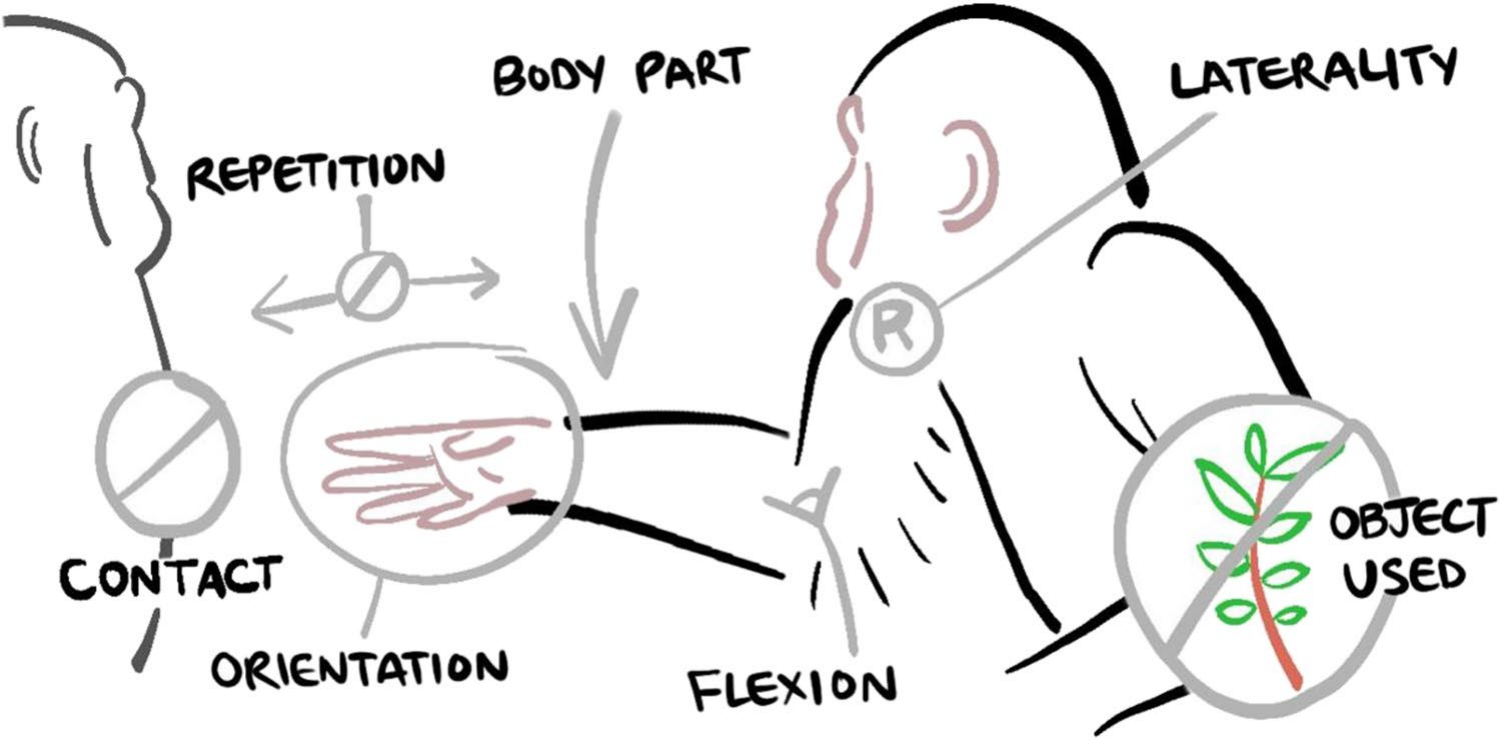
Gesture record: coding the gesture action and its modifiers – example: ‘reach’. *Note.* Bottom-up gesture type construction: coding the gesture action and the modifiers that describe the physical production of the gesture action in more detail taking the illustrated ‘reach’ instance as an example. Gesture record (parent annotation): which bodily movement is performed (*here*: ‘Reach’)? Body part signaller: which body part was used (*here*: ‘Arm’)? Signaller laterality: was it their left or right body part (*here*: ‘Right’)? Object Used: was the gesture produced using an object (*here*: ‘None’)? Flexion: in a free limb gesture action (one in which there is no required contact with an object, substrate, or additional body part in order to perform the action), we consider whether the elbow, wrist, and/or fingers were bent past 45° (*here*: ‘Elbow’)? Orientation: in a free limb gesture action, what direction does the palm of the hand or sole of the foot face (*here*: ‘Side’)? Repetition count: for gesture types that have rhythmic repeated movements, how many times was the action repeated (*here*: ‘No value’ as the ‘reach’ is not a repetition gesture action. Note that this tier is a *free text* variable.)? Body part contact: for contact gestures, we also code the recipient body part that the gesture contacts (*here*: ‘None’ as ‘reach’ is not a contact gesture action). Additionally, we estimate the *audibility* of the gesture instance and whether there was evidence of *directionality*, e.g., whether the reach was extended towards an individual or location of potential interest (Note: both variables not illustrated here). See the controlled vocabulary excel file (GOv1.0_Elan_controlled_vocabulary.xlsx) for full lists of options for the gesture action and modifier variables and the GesturalOrigins coding Protocol (GOv1.0_Protocol) for details on how to code each variable (both files are accessible here: https://github.com/CharlotteGrund/Gestural_Origins_Coding‐methods_paper)

The foundation for describing gestural units in our coding is the *gesture action*, the bodily movement that describes the current gesture instance (e.g., a ‘reach’ as a coordinated extension towards a recipient vs. a ‘beckon’ as a scooping movement away from and back to the signaller, see ESM: GOv1.0_Gesture_action_definitions.xlsx for a full list of gesture actions and their definitions). We then code several variables as ‘child’ tiers of the main gesture action (i.e., variables linked to the gesture record annotation, the ‘parent’ tier) that describe the physical production of the focal gesture instance in more detail (see Fig. 1 below for the main variables coded for each gesture action instance):

Thus, each gesture instance has the action movement at its core together with additional characteristics (‘modifiers’), that can be used as building blocks to further specify the observed behaviour, allowing for flexibility in constructing gesture ‘types’ (and resulting repertoires) at different levels of resolution post-coding. We describe the resulting gesture forms, the non-random combination of gesture actions and their modifiers (see Fig. 1), as ‘morphs’ with no *a priori* assumption about whether or not these reflect a particular ‘type’ or other category of gesture. The creation of gesture ‘morphs’ can either be done manually (as was done previously, although without the flexibility and transparency of the current approach), or through the application of new computational methods to coded gesture data (e.g., clustering algorithms; Mielke et al. in prep). Similar machine learning methods have been used to describe vocal repertoires (e.g., Keen et al., 2021), detecting potentially meaningful higher-order structural patterns in a species’ communicative behaviour that we as human observers (only being able to look at each gesture instance at a time) might otherwise have missed (Wadewitz et al., 2015). While we believe that many of the gesture actions we describe (here and in our supplementary material GOv1.0_Gesture_action_definitions.xlsx), are transferable to non-ape species, and in particular to other primate species, some are species typical. Researchers have the option to easily adjust the gesture action list to account for any species-typical gestural movements (as is the case for any other “controlled vocabulary” presented; for example, our research group has recently adapted this scheme for use in African savannah elephants – some of the adaptations included the addition of ears and trunk to the body parts list).

### Gesture duration and informative action units

Describing the gesture types (and the repertoires that they constitute) is just a first, if essential, step in describing how gestures function and operate in communicative interactions. We consider each gesture instance in a communication event as an element that carries some form of ‘information’ that the signaller wants to convey to the recipient (potentially altered and/or specified in conjunction with other factors such as the context in which it is produced, the signaller/recipient relationship, or its combination with other communicative acts; Graham et al., 2022). With this we mean that there is information about the intended goal in the signaller’s gesture action itself, a reason why the signaller chose this particular gesture action for this particular communicative goal in this particular situation, and that there are various other sources of ‘information’ inherent to the situation (e.g., the context or the signaller’s rank) that may alter/add to the information carried in the gesture action itself. But what is the unit of the gesture instance itself that carries information?

Studies of human co-speech gesture discriminate four stages of a gesture: preparation, action stroke, hold, and recovery (Kendon, 2004). There are potentially important distinctions in the information contained within the action movement itself (action phase) and the *continuation* of the action (hold or repetition phase). In a communication, the action phase (e.g., extending the arm towards the recipient in a ‘reach’) might be sufficient for the recipient to understand the gesture, whereas the (optional) hold/repetition phase (i.e., keeping the reach gesture action in place) may add other information, for example, about the signaller’s willingness to wait or persist. With this framework in mind we differentiate two broad categories of gesture action types: those that incorporate an optional hold/repetition phase (e.g., as in the gesture actions ‘reach’ or ‘raise’ (optional hold phase) or ‘hit’ or ‘stomp’ (optional repetition phase) – we term these *variable gesture action types*) and those without the option for the action to be held or continued in any instance of production (e.g., as in the gesture actions ‘beckon’ or ‘fling’ that always move from the end of action stroke itself directly into the recovery phase – we term these *stable gesture action types*).

**Fig. 2:**
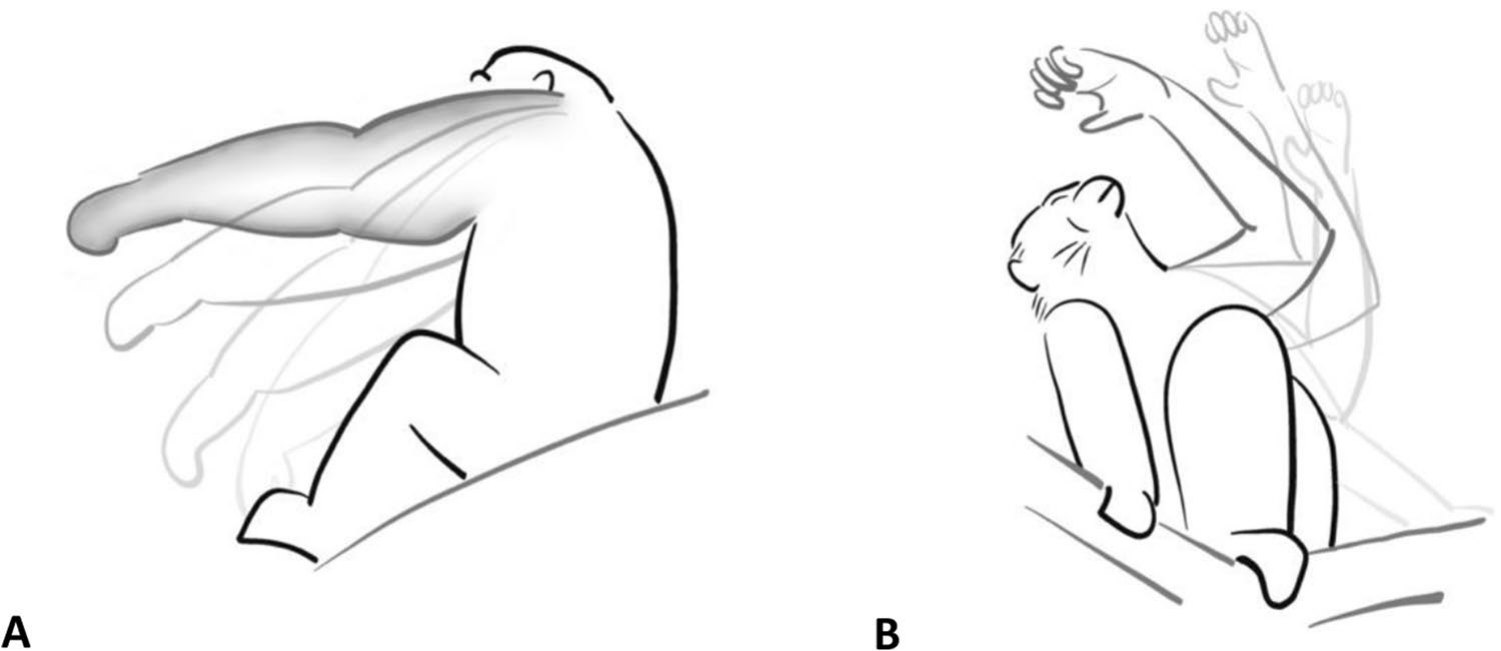
Illustration of the different stroke phases of variable and stable gesture action types using ‘reach’ and ‘beckon’ as examples. *Note.* We assume that the information content within a gesture action may not be evenly spread over the whole gesture duration but may take more the form of discrete units (e.g., action and hold) and illustrate this with two example gesture actions. **A** ‘Reach’ (a *variable gesture action type*): the minimum action unit (MAU) and the gesture action (GA) start as soon as the signaller moves his arm from the neutral position (*very light grey line*), i.e., starts to gesture. The MAU ends when the movement phase is completed, i.e., the reach is in its maximal extension towards the recipient (*dark grey line*; MAU duration = neutral position to MAU end). The gesture action continues until the signaller starts to lower their arm, i.e., when the gesture is not in place anymore. This optional hold phase between the end of the MAU and the end of the GA is indicated by the grey airbrush fill (Gesture action duration = neutral position to GA end, i.e., end of hold/repetition phase). We also annotate when the arm is back in its neutral position to track the total time invested in the gesture production (Full gesture duration = neutral position to neutral position). **B** ‘Beckon’ (a *stable gesture action type*): the MAU and the GA start as soon as the signaller moves his arm from the neutral position (*very light grey line*), i.e., starts to gesture. The MAU ends when the movement phase is completed (i.e., after the full scooping beckon action). As this is a stable gesture action, there is no optional hold/repetition phase, and the gesture action ends at the same time as the MAU (MAU/GA duration = neutral position to MAU/GA end). As in the reach, we also annotate the time when the arm is back in its neutral position (Gesture duration = neutral position to neutral position)

Figure 2 illustrates how we conceptualise the different action phases of A) variable (exemplified by the gesture action ‘reach’) and B) stable (exemplified by the gesture action ‘beckon’) gesture action *types* in our coding scheme. Like Kendon’s (2004) preparation and action stroke, we differentiate a part of the gesture action that should contain the minimal information necessary to discriminate it from any other gesture action, which we term the *minimum action unit* (MAU). The MAU of every gesture action type (coded once for every gesture instance) starts at the point when the signaller starts to gesture, i.e., moves the body part (here arm) out of its communicatively ‘neutral’ state (lightest grey line in Fig. 2) and uses it to perform the gestural movement. We define a communicative ‘neutral’ state when the body part employed in the gesture is at rest or used in non-communicative actions (e.g., feeding). The MAU ends when the gesture action is fully in place (darkest grey line in Fig. 2). For gesture action types that have the option to be held in place (e.g., ‘reaches’, Fig. 2A) or to be (rhythmically) repeated (e.g., ‘hitting’), i.e., *variable gesture action types*, the gesture action itself does not necessarily end with the MAU but may continue until the signaller decides to stop displaying the gesture and returns the respective body part back into a (communicatively) neutral position (e.g., retracting his arm back to a resting position, or starts to use it for locomotion, or feeding, etc.) or alternatively, uses it to perform the next gesture action. The optional hold/repetition phase is indicated by the grey airbrush fill in Fig. 2A. We call the duration from the start of the first movement from the neutral position to the end of the gesture action the *gesture action duration.* Here we further differentiate between the gesture action duration of a *continued gesture action instance* (an instance of variable gesture action type production where the signaller did make use of the optional hold/repetition phase) and the gesture action duration of a *minimised gesture action instance* (an instance of variable gesture action type production where the signaller either did not make use of the optional hold/repetition phase or the instance belongs to a stable gesture action type). As a result, in *stable gesture action types* the gesture action typically ends at the same time as the MAU (i.e., gesture action end = MAU end, as they do not have a hold/repetition phase: e.g., ‘beckon’ in Fig. 2B), whereas in the *variable gesture action types* the gesture action may or may not end at the same time as the MAU, depending on its specific use in the current communication (ESM: GOv1.0_Gesture_action_definitions.xlsx in the supplementary material provides detailed descriptions of identified gesture actions and their respective gesture action phases). In some gesture action types, duration itself has been used to discriminate different categories of gesture action (for example, touch and touch-long). While these are *a priori* decisions (based on patterns of use established in previous research), these are also aspects that can be tested in the future. For example, by lumping all cases of touch actions, and plotting the duration of different aspects of the gesture actions to explore whether there is a case for apparent categories within them (for example, these might be illustrated through a bi- or multi-modal distribution of durations, as opposed to a normal one). Our coding also allows for durations to be marked as unknown where it is not possible to establish the start and/or end of a phase, providing the option for these cases to be easily excluded from analyses.

Considering where and how information is encoded within gestural signals is fundamental to investigating how they operate in the communicative system. In our coding, we thus measure four different points in the gesture instance: the start of gesturing (Gesture start time), the end of the minimum action unit (MAU end time), the end of the gesture action (Gesture action end time, in case of stable gesture action types this is always the same as the MAU end time; in case of variable gesture action types it depends on the instance of use: either at the end of the MAU (when no hold/repetition phase was included) or at the end of the hold/repetition phase), and when the gesturing body part is back in its neutral position or starts to produce a new gesture action (Gesture end time). From these we can calculate (post-coding) the *full gesture duration* (all movement involved to produce the gesture instance, including its recovery: neutral position to neutral position), the *gesture action duration* (the movement of the gesture action and (optionally) it being held in place: neutral position to gesture action end time) and the *MAU duration* (neutral position to MAU end time), the part of the gesture action that contains the information for the gesture instance to be “understood” as being a particular gesture action or morph.

Given that the MAU is the section of the gesture that leads to it being a recognised unit by the recipient, it is likely subject to physiological and selective pressures for efficient communication, for example compression (Heesen et al., 2019; Safryghin et al., 2022), and we expect it to be quite consistently expressed across instances. In contrast, if apes incorporate the use of an optional ‘hold’ phase in their gestures, we predict that these show greater variation in duration: production of a hold is more likely to depend on variables related to the specific instance of communication, such as the latency for the recipient to respond to the gesture or the signaller’s willingness to invest in a particular outcome.

#### Worked example 1: Gesture durations

**Fig. 3:**
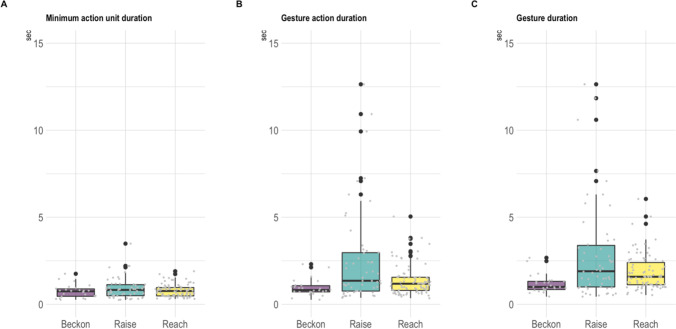
Worked example 1: Difference in variability between the MAU duration, the gesture action duration, and the full gesture duration. *Note*. Boxplots of the gesture actions ‘beckon’ (*n* = 26), ‘raise’ (*n* = 54), and ‘reach’ (*n* = 85) showing the difference in duration variability between the **A** *Minimum action unit (MAU)* phase of a gesture action (*MAU duration* median for ‘beckon’ = 0.74 s (range = 0.26–1.75 s); for ‘raise’ = 0.82 s (range = 0.25–3.48 s); and for ‘reach’ = 0.76 s (range = 0.30–1.90 s; *MAU duration* mean for ‘beckon’: 0.74 s (SD = 0.37); for ‘raise’ = 0.91 s (SD = 0.60); and for ‘reach’ = 0.79 s (SD = 0.35)), **B** the time taken to produce and maintain the gesture action (*Gesture action (GA)* phase of a gesture action*:* *GA duration* median for ‘beckon’ = 0.81 s (range = 0.26–2.3 s); for ‘raise’ = 1.34 s (range = 0.37–12.64 s); and for ‘reach’ = 1.18 s (range = 0.35–5.04 s); *GA duration* mean for ‘beckon’: 0.95 s (SD = 0.48); for ‘raise’ = 2.55 s (SD = 2.75); and for ‘reach’ = 1.36 s (SD = 0.87)) and **C** including the recovery phase of the gesture (*Full gesture duration* median for ‘beckon’ = 1.00 s (range = 0.44–2.67 s); for ‘raise’ = 1.90 s (range = 0.44–12.64 s); and for ‘reach’ = 1.58 s (range = 0.53–6.10 s); *Full gesture duration* mean for ‘beckon’: 1.17 s (SD = 0.51); for ‘raise’ = 2.87 s (SD = 2.82); and for ‘reach’ = 1.84 s (SD = 1.02)). Species = *Gorilla beringei beringei; n* = 165 instances of gesture use, *n* = 26 signallers, filtered from data of mountain gorilla gestural behaviour collected on four social units (Mukiza, Oruzogo, Kyagurilo, Bitukura) in Bwindi Impenetrable National Park, Uganda, between 2019-2022 – data in ESM: Worked_example_1_data.csv)

Here we provide a worked example considering gesture duration in different ways. We examine the *MAU durations* of three common gesture actions in mountain gorillas (‘beckon’, ‘raise’ and ‘reach’; Grund et al., in prep) and compare them to their respective *gesture action* and *full gesture duration* (see Fig. 3). We expected the gesture action durations to be more variable for the gesture actions ‘raise’ and ‘reach’ (as they have an optional hold phase) compared to the more stable ‘beckon’ gesture action. To assess reliability of the results we conducted single-score intra-class correlation (ICC) tests (ICC2 tests, model = ‘two-way’, type = ‘agreement’, *psych* package, v2.2.9, Revelle, 2022) on the measurements ‘MAU duration’ and ‘Gesture action duration’ (using the timings G_start_T, MAU_end_T and GA_end_T, see ESM IRR_test.docx for more details) of a small subset (*n* = 40 communications) of the latency dataset (*n* = 250 communications, see worked example 2). The overall agreement between the two coders (CG, CH) was good for the MAU duration (ICC = .97, 95% CI from .95 to .99, F(39, 39) = 73, *p* < 0.01) and excellent for the Gesture action duration (ICC = 0.99, 95% CI from .99 to .99, F(39, 39) = 7796, *p* < 0.01). The was no consistent coding bias observed (see ESM_IRR_test.docx in ESM for graphs showing the coding differences and more details on the test and the dataset used).

As predicted, within the gesture actions ‘raise’ and ‘reach’ there is more variability in the duration of the whole gesture (see Fig. 3B: *gesture action duration* and 3C: *full gesture duration*) as compared to their respective MAUs (see Fig. 3A: *Minimum action unit duration*). Interestingly, the duration of the recovery phase (the time from the end of the gesture action to when the body part returns to rest) has much less impact, as seen by the smaller difference between *gesture action duration* and *full gesture duration*, thus the variation between instances of gesture expression from within a gesture type seems to be focused within the hold phase. As predicted, this pattern seems to vary between gesture action types, with less variation in duration between gesture action and minimum action unit for the stable gesture action ‘beckon’.

The fact that the minimum action unit seems to be consistently shorter and less variable in length than the gesture action (with differences in the degree of that trend between gesture actions) underlines the importance of making this distinction in the first place. Importantly, at the end point of the MAU the signaller has already communicated the core information about the gesture action to the recipient, which also has important implications on how we might want to investigate sequence structures and calculate response latencies (see next section).

### Communication structure and latencies: Interpreting gesture sequences and response waiting

Many communications can be described as relatively simple behavioural strings – the signalling individual looks at the recipient, gestures once, pauses, and the recipient reacts with an apparently satisfactory response (ASO) and the communication ends. However, some communications appear structurally more complex: for example, signallers may deploy several gestures (of the same or different types) consecutively (and/or overlappingly). In communications with several gesture instances, one gesture may be directly followed by another with little or no time lapse in between or, alternatively, the signaller may only gesture again after a longer pause, during which they continue to monitor the recipient.

One interpretation of these structures is that in the former case, gestures are produced as rapid sequences combined independently of the recipient’s behaviour, whereas in the latter, gestures are separated by *response waiting* by the signaller with subsequent gestures representing persistence after the failure of the earlier request (Liebal et al., 2004; Hobaiter & Byrne, 2011b; McCarthy et al., 2013). In most approaches, researchers take an *a priori* decision to categorize gestures into sequences based on the amount of time between them. In some cases, these are dependent on a set time cut-off between gestures alone (Liebal et al., 2004; McCarthy et al., 2013), while others ground grouping decisions on a more theoretical framework. i.e., combining time-based cut offs with the presence or absence of other behaviour in the interim (e.g., Hobaiter & Byrne, 2011b). For example, we and others have used an (*a priori*) 1-s minimum interval between the end point of one gesture and the start of the next, to indicate that two gesture instances belong to different sequences, i.e., that they are two instances of gesture with response waiting in-between them (Hobaiter & Byrne, 2011b; Luef & Liebal, 2012; Heesen et al., 2019).

Where response waiting is considered present, researchers interpret the next gesture in line as ‘persistence’ (same gesture type) or ‘persistence with elaboration’ (new gesture types) towards achieving the same goal rather than the production of a new communication (e.g., Cartmill & Byrne, 2007; Leavens et al., 2005). Describing different types of gesture sequence, for example, the addition of subsequent gestures as part of the same communication, as persistence towards the same goal, or as a new communication, is helpful in discriminating the different types of communicative structure in ape gesture. However, within non-human gesture, primate research is strongly biased to great apes, particularly chimpanzees (Rodrigues et al., 2021), which means that the traditional application of rules is based on time-intervals or behavioural indicators that were shaped through the study of a relatively small number of chimpanzee populations and that may not always be appropriate when considering gesture in other ape species such as mountain gorillas, or orang-utans, let alone non-ape species (Farrar et al., 2021). Even the closely related bonobos seem to show different tendencies in response waiting times (Fröhlich et al., 2016). Rigid time-based or behaviour-based cut-offs to describing gestural structures, such as sequences, may thus be inherently problematic when trying to extend gesture research to other species or populations.

**Fig. 4:**
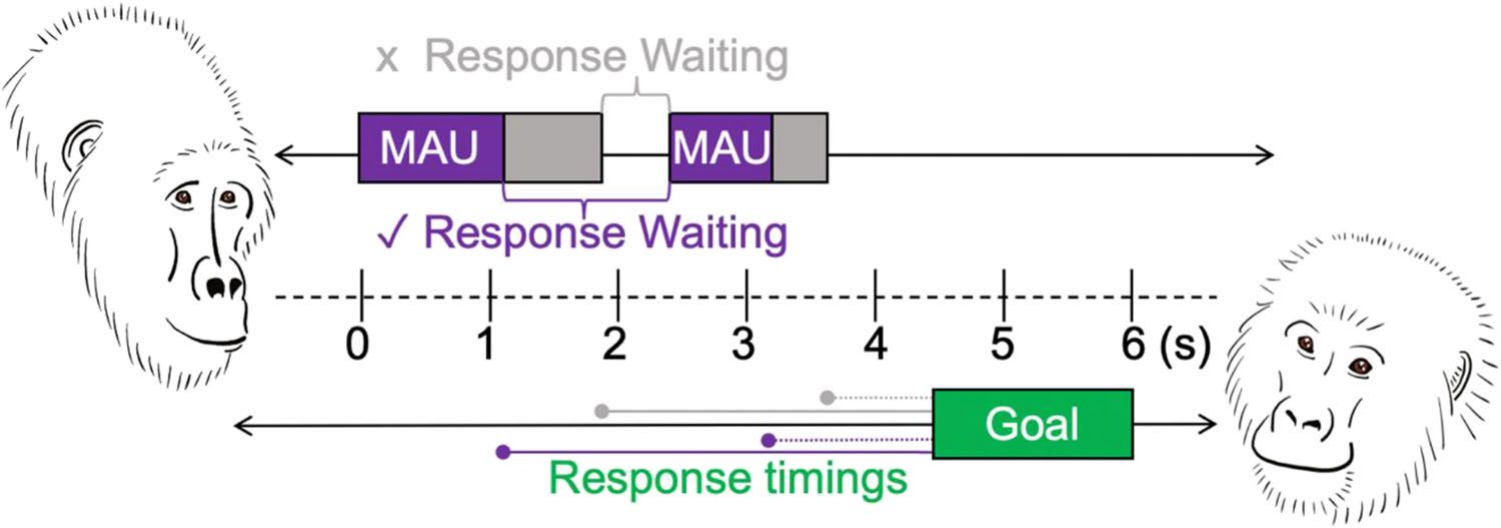
Visualisation of different ways in which response latencies and inter-gesture intervals can be measured using the GesturalOrigins coding scheme. *Note.* Each communication can be viewed as a string of behaviours on a timeline that starts with the gesturing of one individual towards another and ends with a particular behavioural outcome (typically, either the goal of the signalling individual (ASO) or the failure of the communication). In this example of a simple gestural communication, individual A (the signaller) on the left gestures twice and individual B (the recipient) responds with an apparently satisfactory outcome (the ‘goal’ of the communication) after a certain amount of time has elapsed. If we take the inter-gesture interval as starting at the end of the gesture action duration and ending at the onset of the next gesture, it is < 1 s and would not meet the time-based cut off for response-waiting to be present and both gestures would be considered part of the same sequence. However, when investigating inter-gesture intervals and response-waiting times the end of the MAU may be of particular interest, because it approximates the point in time where all the necessary information for the gesture action to be understood as a case of this particular gesture action should be in place. If we consider the inter-gesture interval as starting at the end of the MAU and ending at the onset of the next gesture, the time-interval in this example is then > 1 s and would meet the time-based cut off for response-waiting, and the two gesture instances would not be part of the same sequence. Apart from inter-gesture intervals the coding scheme offers great flexibility in calculating recipient response latencies (see worked example 2). Some of them are indicated with purple and grey lines leading from different gesture end points to the *Outcome* (*green goal box*). Note that for the purpose of clarity and regarding the fact that the difference between gesture action duration and full gesture duration seems consistent and small (see worked example 1) no distinction is made between these two durations in the graph

It is also important to recognise that the length of the interval between any two gestures (and with it the potential for reaching a time-based cut-off) depends on which point in the gesture action phase one considers a suitable starting point for the onset of response waiting. Figure 4 below is a schematic illustration of a (simple) communication where one signaller (A) gestures twice towards the recipient (B) and the latter responds with an ASO (i.e., ‘goal’) after a particular time has elapsed.

If for example a ‘reach’ gesture is produced with an extension of the arm, and then that reach is held in place for several seconds – response waiting could be considered to start a) once the initial movement necessary to produce the gesture action is completed (i.e., at the MAU end point, the end of the extension movement – Fig. 4, purple colour) or b) when the gesture action is completed and the limb is returned to rest or starts to produce the next gesture action (so at the end of the hold phase, indicated in Fig. 4 by the grey colour, ‘traditional’ approach). If we require that signallers show at least 1 s of pause, or a behavioural indicator such as a visual check to mark response-waiting as present, then its presence will vary depending on whether we measure from point a) or b). The data illustrated in worked example 1, support the suggestion that the information content within some gesture actions may not be evenly spread over the *full gesture duration* and it may be appropriate to consider the point at the end of the *MAU* (so when the signaller has communicated the core information in the action phase) as the start of response waiting.

What about the end points of response waiting? From the perspective of the whole communication (and the fact that we assume a global goal), response waiting may only be considered to be over when the recipient changes their behaviour in the desired way and the signaller’s intended goal (i.e., the reason for her gesturing in the first place) has been met. With the *Outcome time* variable, we mark the time point when the goal is fulfilled, and the communication has ended. Whenever the *Outcome* cannot be determined (e.g., in cases of communicative failure) it is marked as ‘unknown’ (but see ESM: GOv1.0_Elan_controlled_vocabulary.xlsx – Sheet ‘Goal’ for a list of identified signaller goals). The *Outcome time* variable together with the *MAU* and *gesture action* timings of the gesture instances allows us to calculate different response waiting times, e.g., ones from the perspective of the whole communication that may not stop at the start of the next gesture but that extend from the current gesture instance to the actual end of the communication, or ones that, for example, only consider the final gesture in the communication as relevant when calculating recipient response latencies (as e.g., in worked example 2). As the signaller tries to alter the recipient’s behaviour throughout the communication and is likely to adjust her gesturing to the behaviour of the recipient (or negotiates, through gestural exchanges with the recipient), it may also be interesting to explore recipient responses (latencies) prior to any (satisfactory) communicative outcome. We therefore also code the following recipient timings on the level of the communication: did the recipient respond with a gesture (*Gesture recipient*, ESM: GOv1.0_Protocol – section 2.13) or a vocalisation (*Vocalisation recipient*, ESM: GOv1.0_Protocol – section 2.14) at some point during the communication? Did the recipient react to the gesturing in some form behaviourally (*Behavioural Change* 1 and 2, ESM: GOv1.0_Protocol – sections 2.17 and 2.18) prior to the end of the communication?

With the GesturalOrigins scheme, researchers can calculate inter-gesture intervals and recipient response latencies from different gestural end points (e.g., *MAU end* or *Gesture action end*) flexibly and investigate their effects on interactional dynamics in the communication. Our next worked example shows why high-resolution coding in time-sensitive data and a data-driven approach to the study of sequence structure and latencies may be crucial when extending the study of gesture to new species.

#### Worked example 2: How long is long enough for response waiting

**Fig. 5:**
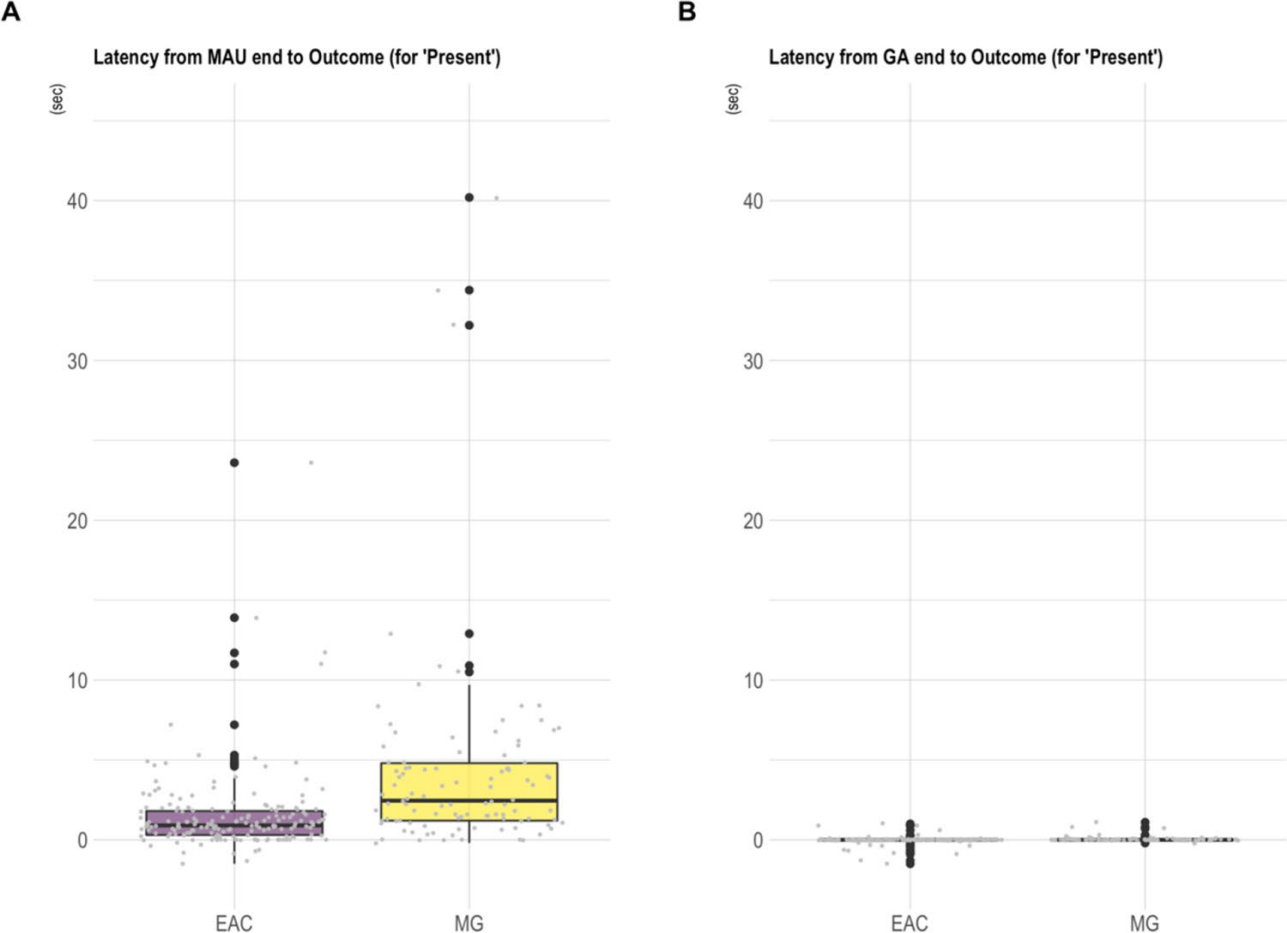
Worked example 2.1: Mountain gorilla and chimpanzee recipient latencies to (behaviourally) respond to a signaller’s request to be groomed using the ‘present’ gesture action (*n* = 281 successful communications). *Note*. Boxplots showing the response waiting times (latencies to respond) in East African chimpanzees (EAC; *n* = 177 communications, *n* = 70 signallers) and mountain gorillas (MG; *n* = 104 communications, *n* = 27 signallers) for the gesture action ‘present’ and the outcome ‘grooming’ (total: *n* = 281 communications) when considering either **A** the *minimum action unit (MAU)* end point or **B** the *gesture action (GA)* end point as the start of response waiting (see Fig. 4 for a conceptual visualisation of different response latency measurements). Mountain gorillas seem to be slower to respond to grooming requests than East African chimpanzees. Note that a single value (the maximum value of 78.1 s for the mountain gorillas) is not visually represented on the graph (though not omitted from the scaling) for the purpose of better resolution. As the ‘present’ gesture action has the characteristic of being held in place until the goal is fulfilled (in successful communications) there is unsurprisingly little variation in the gesture action to outcome latency (graph B), which is close to 0 in both species (MG *GA.Outcome* latency range = – 0.2–1.1; EAC *GA.Outcome* latency range = – 1.5–1.0 s). The negative latencies result from instances where the recipient already responded to the gesture before the respective action phase (MAU and/or GA) was completed

Using gestural data from East African chimpanzees (two communities: Sonso and Waibira, Budongo Forest, Uganda) and mountain gorillas (four social units: Mukiza, Oruzogo, Kyagurilo and Bitukura, Bwindi Impenetrable National Park, Uganda) we investigated two different time measurements in 957 successful communications (mountain gorillas: *n* = 250 communications, *n* = 37 signallers; chimpanzees: *n* = 707 communications, *n* = 115 signallers). We calculated the latency both from a) the end of the MAU to the outcome (*MAU.Outcome latency*, i.e., from the point the gesture action phase was completed until the goal was fulfilled, see purple dotted-line in Fig. 4) and then from b) the end of the whole gesture action to the outcome (*GA.Outcome latency*, i.e., from when the signaller stopped displaying the gesture until the goal was fulfilled, see grey dotted-line in Fig. 4). Where there were several gestures in the communication, we only considered the MAU or GA of the final gesture (the one closest to the outcome). Figure 5, plots A and B show the data for a single gesture action (‘present’) commonly used in both species for the initiation of ‘grooming’ (*n* = 281 successful communications, data (ESM: Worked_example_2.1_data.csv) only includes presents for grooming to control for possible variation due to the goal of the signaller).

The data shown in the graphs indicate a broad tendency for mountain gorillas to take longer to respond to presents for grooming than chimpanzees (Mountain gorilla *MAU.Outcome latency*: range = – 0.72–78.1 s, median = 2.45 s, mean = 4.9 s (SD = 9.48), *n* = 104 communications; East African chimpanzee *MAU.Outcome latency*: range = – 1.5–23.6 s, median = 0.09 s, mean = 1.52 s (SD = 2.56), *n* = 177 communications).

**Fig. 6:**
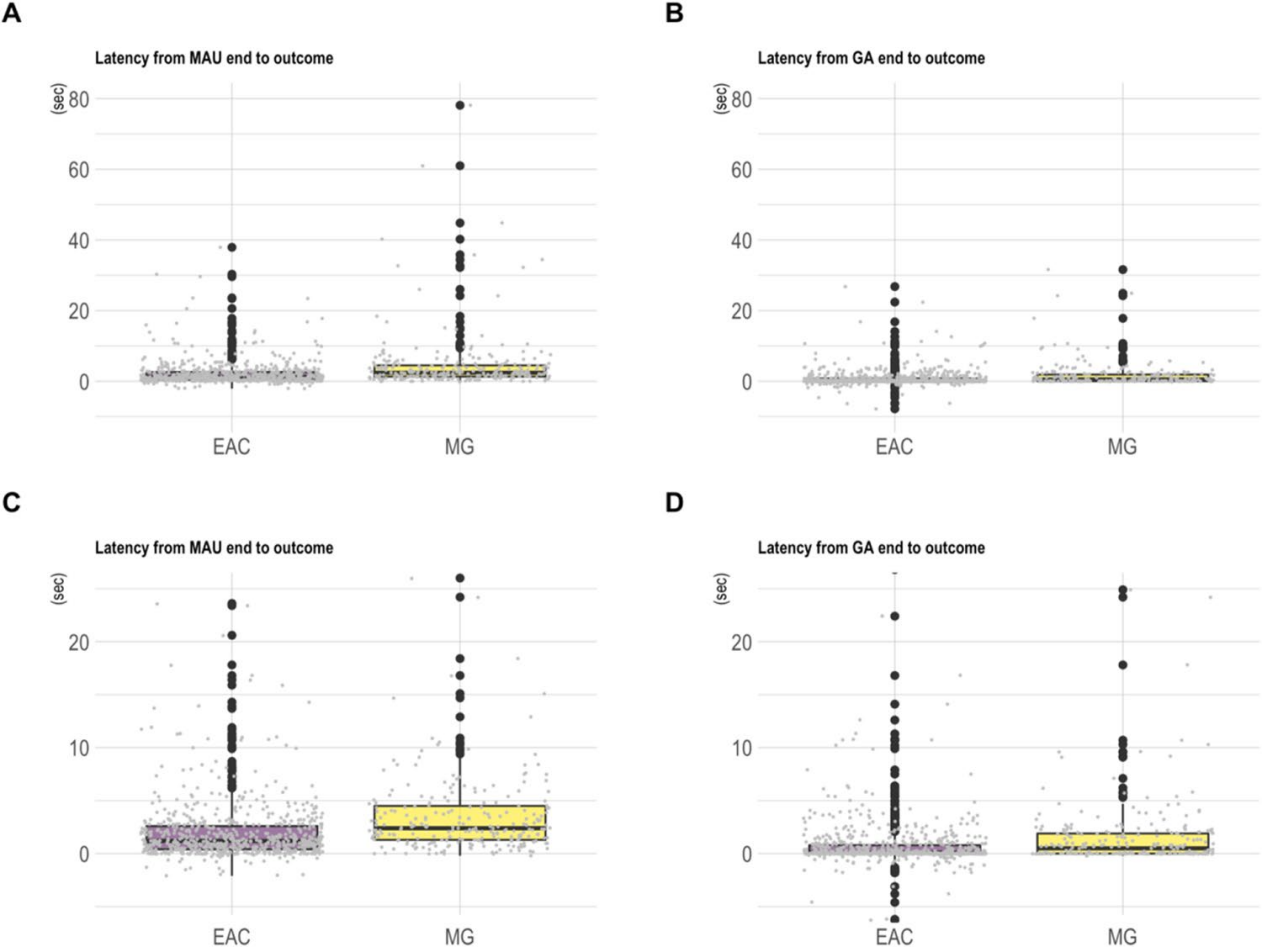
Worked example 2.2: Mountain gorilla and chimpanzee recipient latencies to (behaviourally) respond to a signaller’s gestures (successful communications, all goals except ‘play’). *Note*. Boxplots showing the response waiting times (latencies to respond) in East African chimpanzees (EAC) and mountain gorillas (MG) in 958 successful communications (MG: *n* = 250 communications, *n* = 37 signallers; EAC: *n* = 707 communications, *n* = 115 signallers – including all gesture actions, excluding the goal ‘play’ – data in ESM: Worked_example_2.2_data.csv) when considering either the MAU end point (**A** and **C**) or the gesture action end point (**B** and **D**) as starts of response waiting. The graph **A** includes the full range of latency values observed for MAU end to Outcome (range: – 2.1–78.1) and graph B the full range of latency values for Gesture action end to Outcome (range: – 7.8–31.6) while the graphs **C** and **D** show only those latencies with values between – 5 and 25 s (for a better resolution on where the majority of the data lies, while not omitting the more extreme values from the scaling). The data suggest that mountain gorillas take longer to respond to gestural requests as compared to chimpanzees when considering the MAU end point as the start of response waiting as well as when considering the GA end point as the start of response waiting. As in worked example 2.1, the negative latencies result from instances where the recipient already responded to the gesture before the respective action phase (MAU and/or GA) was completed

To see whether this apparent species difference in gesturing is observed more globally, we plotted data for all gesture actions (and all goals excluding ‘play’; Fig. 6A–D; data in ESM: Worked_example_2.2_data.csv).

Though preliminary, Figs. 5 and 6 together suggest potentially noteworthy species-differences in interactional dynamics between chimpanzees and mountain gorillas. Most obviously, a slower recipient responsiveness to gestures in mountain gorillas (e.g., the larger average *MAU.Outcome* latency values in MG (mean = 4.73 s (SD = 8.55), *n* = 250) compared to EAC (mean = 2.08 s (SD = 3.46), *n* = 707, see Fig. 6). And second, while both species show a clear (and quite similar) relative difference between *Gesture action duration* and *MAU duration*, mountain gorillas show potentially greater variability in latency when measured from either the end of the MAU or the end of the Gesture action (divergence in values between *MAU.Outcome* and *GA.Outcome* latency in EAC (MAU Outcome latency mean = 2.08 s (SD = 3.46) vs. GA.Outcome latency mean = 0.71 s (SD = 2.25); *n* = 707); and in MG (MAU Outcome latency mean = 4.73 s (SD = 8.55) vs. GA.Outcome latency mean = 1.58 s (SD = 3.56); *n* = 250).

The data presented here (mountain gorillas *n* = 250 communications; chimpanzees *n* = 707 communications) were coded by three different researchers (AS, CG, GB) and species differences could be the result of coding biases (between CG and AS/GB). To have a measurement of the reliability of the results CG (mountain gorilla coding) coded subsets of the chimpanzee dataset (*Sonso*: 25/555 communications coded by AS; *Waibira*: 20/152 communications coded by GB) and conducted single-score intra-class correlation (ICC) tests (ICC2 tests, model = ‘two-way’, type = ‘agreement’, *psych* package, v2.2.9, Revelle, 2022) on the durations *MAU.Outcome* latency and *GA.Outcome* latency as well as the time measurements used to calculate them (see ESM: IRR_test for more details on ICC2 tests looking at MAU_end_T, GA_end_T and Outcome_T separately). Overall, there was good agreement between AS and CG (MAU.Outcome latency ICC = .92, 95% CI from .79 to .97, F(24, 24) = 29, *p* < 0.01); GA.Outcome latency ICC = 0.95, 95% CI from .88 to .98, F(24, 24) = 50, *p* < 0.01) and between GB and CG (MAU.Outcome latency ICC = .95, 95% CI from .85 to .98, F(19, 19) = 45, *p* < 0.01; GA.Outcome latency ICC = .98, 95% CI from .95 to .99, F(19, 19) = 88, *p* < 0.01) in coding the latency variables. In plots of the deviations from the mean for the MAU.Outcome and GA.Outcome latency variables, visual inspection suggested a possible bias towards longer latencies in CG as compared to AS (mean coding difference MAU.Outcome latency = 0.48; mean coding difference GA.Outcome latency = 0.38, most likely due to consistent differences in coding the Outcome time variable, see ESM: IRR_test.docx, Figure ESM.2 for more details). To assess whether any species differences could be explained by a coder bias, we added the mean coding difference between the two coders (CG and AS) to all of AS latency values. We continued to find a consistent difference in latencies between the species (see ESM: IRR_test.docx, Figure ESM.4).

The worked examples we present here serve to illustrate potential types of analyses that our coding scheme offers. A proper analysis of inter-species differences in, for example, latencies to respond would have to carefully consider other sources of variation, for example, individual and/or group-level differences. One issue in our approach that we would like to highlight is that – as they are a new concept – we defined the MAUs for each gesture action *a priori* using the minimal information we as human observers need to have available to be able to distinguish between the gesture actions (see definitions in sheet GOv1.0_Gesture_action_definitions.xlsx). The parts of the gestural movement that we consider crucial may not always reflect the minimal information necessary for the apes to discriminate one gesture action from the other. These definitions will benefit from being tested with detailed coded data in the future. One potential test of their validity for a particular gesture action, may be to use the latencies to respond. Here, where the MAUs of a gesture action appear to be consistently understood “too early” across uses (i.e., the time to respond is shorter than the MAU – represented by negative MAU latencies to the outcome), irrespective of goal or interacting individuals, it may be appropriate to reconsider the definition of that MAU. On the other hand, if we don’t find consistent negative MAU latencies to the outcome, we can be more certain that we at least did not overestimate the part of the gestural movement that encodes important information.

### Multisignal combinations: integrating facial expressions and vocalisations

Gesture research has been criticised for neglecting other communicative elements such as vocalisations or facial expressions, typically by excluding them from data collection. Studying gesture in isolation, rather than considering communication as a whole, limits our interpretation of other species’ communicative capacities (e.g., Slocombe et al., 2011; Genty, 2019; Liebal et al., 2022). At the same time, coding is an extremely time-intensive undertaking and there is always a trade-off between coding effort, coding reliability, and information yield. Reliable approaches to holistic coding may benefit from the use of multiple synchronised cameras to allow for capturing the signaller (and ideally also the recipient) from multiple angles, ensuring the detection of the full range of different signal types. But doing so dramatically increases the investment in both video data collection and in subsequent data coding. In practice, while holistic coding may be ideal wherever possible, sacrifices are required. For example: in our current work, we do not incorporate continuous coding of vocal and facial signals, and instead mark them only when they occur in conjunction with gesturing. In this way, whenever there is a gesture instance, non-gesture signals that overlap in time with gestures are captured and their potential communicative function can be investigated. However, the GesturalOrigins template is adjustable to specific projects’ needs, and includes tiers that provide users with the option to code vocal and facial signals continuously, throughout the full communication. It is also straightforward to add new tiers that further specify detailed coding of additional signals or incorporate other software for specific research questions. For example: Elan has a built-in option to employ “Praat” (Boersma & Weenink, 2022), an opensource software for acoustic analysis.

### Potential influence of socio-ecological factors on gesturing

There is a growing understanding of the importance of pragmatics in primate communication (Seyfarth & Cheney, 2018; Arnold & Bar-On, 2020), for example, recent work in ape gesture has shown that apparent ambiguity in the meaning of some bonobo gestures can be resolved by including information on the behavioural context in which they are produced (Graham et al., 2020). For each communication, we code the interactants’ basic behavioural context prior to the onset of gesturing and after the communication has ended (e.g., ‘resting’, ‘grooming’, ‘sex’; see ESM: GOv1.0_Controlled_vocabulary.xlsx – Sheet ‘Context’ for a list of all behavioural contexts). Similarly, individuals’ use of gestures may vary with their sociality and social relationships (e.g., Bard et al., 2014; Pika & Fröhlich, 2018; Fröhlich et al., 2022; Hobaiter & Byrne, 2011b). The ability to include richer social and environmental information on gesture production thus seems important to further refine our study of its use. Variation in life-history and in the socio-ecological context of production may shape gesture use differently between individuals, communities, and species (Graham et al., 2022). Many aspects, such as individual, species, community size, cohesiveness, age, sex, social rank, or connectedness are independent of the immediate coding of a gesture instance and thus do not need to be directly coded with each instance. However, some aspects of gesture production are specific to a particular communication and in GesturalOrigins we include the ability to code for variables such as the *location* of signaller and recipient (e.g., tree, ground; see ESM: GOv1.0_Protocol – sections 2.47, 2.51) during gesturing, or the *visibility* (see ESM: GOv1.0_Protocol – section 2.53) and spatial *distance* (see ESM: GOv1.0_Protocol – section 2.40) between them.

## Discussion and outlook

The study of gesture, like many aspects of behaviour, benefits substantially from the use of replicable and transparent methods. One means to do so is to employ shared frameworks for coding that allow like-with-like comparison of research within and between research groups. However, in some cases their adoption may prove challenging where different research groups have well-established but variable approaches to coding and describing their data. This variation is not in itself problematic, but it does present a challenge for comparability and replicability. With the GesturalOrigins framework we aim to provide a bottom-up approach to data coding that allows researchers to extract features of interest in highly diverse ways, building gestural units that vary in length or construction and that are deployed with different criteria. Many of the established criteria used in existing gestural research, for example, the use of a particular time-interval to discriminate sequences, can still be extracted. But importantly, these can be varied and – as a result – compared, tested, and validated more easily. Although to do so requires that methods, including definitions of variables (e.g., goals, gesture actions, etc.), are fully described in sufficient detail when publishing (Rodrigues et al., 2021).

The advantage of these new methodological tools is that, in addition to providing enhanced replicability, they allow the exploration of new questions. Using in-video annotation software, we can consider gesture units in increasingly fine-grained detail – for example, distinguishing the parts of a gesture that are necessary for production across all cases (such as the preparation and action stroke) from those that are optional and can vary with each instance of production (such as the hold, repetition, or recovery). In our worked examples, we show that aspects of gesture duration vary in non-trivial ways. Not only are gestures of the same type longer when we consider additional phases of production, but they show substantial variation in length within a phase suggesting that these phases do represent optional additions. A more data-driven understanding of how information is encoded and conveyed in gestural signalling may be crucial for a variety of research questions. For example, recent studies of Zipf’s law of compression – which posits that more frequently used signals are shorter in length (and is widespread across systems of human and non-human communication; Zipf, 1949; Ferrer-i-Cancho et al., 2022; Favaro et al., 2020) – shows only limited evidence in chimpanzee gesture (Heesen et al., 2019). One possible explanation is that, to date, measurement of full gesture durations may be masking its effect (Safryghin et al., 2022). Compression of a signal in an individual’s or species’ repertoire should act on those parts of the signal necessary to every instance of its production (in our framework the MAU), but this pattern may be masked if we consider optional elements of their production that vary in each instance of communication – such as an extended hold of a *reach* when requesting a particularly valuable food item, or the extended repetition of a *shake object* gesture’s action stroke in an important sexual solicitation. Thus, using bottom-up approaches such as GesturalOrigins, we can flexibly extract timings from different points in the gesture action (e.g., at the end of the MAU as well as at the end of the ‘hold’) and test how they may impact sequence structure and shape gestural communication.

Exploring evolutionary hypotheses about the trajectory of gesturing through the hominid lineage requires combined data across diverse species and populations of extant apes. Doing so not only requires truly vast datasets but also that our approach to coding avoids a species-centric bias that may not accurately describe the gesturing of other ape species. At present, ~75% of great ape gesture studies have been conducted on chimpanzees (Rodrigues et al., 2021). Early comparisons already suggest species differences in the expression of gesture forms (e.g., limb use between chimpanzees and orang-utans; Knox et al., 2019) and in responsiveness (chimpanzees and bonobos; Fröhlich et al., 2016). The duration of time intervals that reflect connections or distinctions in strings of behaviour, for example response-waiting, or latency to respond, may also be shaped by species socio-ecology. In our second worked example, we show that mountain gorillas typically show longer latencies to respond to gestures as compared to chimpanzees. Thus, species comparisons using a set interval may not provide a like-with-like comparison of behaviour, and care is needed in the application of baselines established in chimpanzees, to avoid mischaracterising gestural communication in other apes. Of course, our coding scheme also has limitations. For example, behavioural categories need to be established and defined pre-coding, and biases may be introduced at this level. The available options for a particular variable (e.g., the list of contexts, goals, or gesture action types) may be more appropriate for one species than another (e.g., because the latter has not yet been extensively studied) or more detailed for frequently occurring, as compared to rarer, behaviour. However, while there is a cost to re-coding, these can be adjusted as experience of a species or context is gained. Open-access video examples (like the Great Ape Dictionary) may help to improve replicability across studies as researchers may have access to video examples of gesture actions that can be used to determine whether a particular action is present in a specific dataset or species.

Other new and exciting methodological tools are on the horizon, which may increase the efficiency of current tools like our coding scheme. For example, we are starting to delegate some of the more time-intensive aspects of video coding, such as the detection of species (Beery et al., 2019), individuals (Schofield et al., 2019), and behaviour (Bain et al., 2021) to machine learning models. Soon, it may be possible to delegate the detection of social interactions (that may contain gesturing) in large video corpuses such as the Great Ape Video Ark (Hobaiter et al., 2021; Wiltshire et al., in review) to similar automated approaches, and even gestural behaviour itself and the gesture instances coded studiously by hand and eye may eventually be accurately detected with machine learning approaches. While probably not all aspects of coding will easily be automated, for those variables where it is possible, large pre-annotated training sets with accurately coded time-stamped data will be essential to model training. Thus, template-based video coding may also be a facilitator for important methodological milestones in the field of gesture research.

## Data Availability

All data and materials referred to in this article are available on GitHub (https://github.com/CharlotteGrund/Gestural_Origins_Coding-methods_paper) and/or included in this published article (and its supplementary information files).

## References

[CR1] Altmann J (1974). Observational study of behavior: Sampling methods. Behaviour.

[CR2] Arnold K, Bar-On D (2020). Primate pragmatics, expressive behavior, and the evolution of language. Animal Behavior and Cognition.

[CR3] Baker M (2016). Reproducibility crisis. Nature.

[CR4] Bain M, Nagrani A, Schofield D, Berdugo S, Bessa J, Owen J, Zisserman A (2021). Automated audiovisual behavior recognition in wild primates. Science Advances.

[CR5] Bard KA (1992). Intentional behavior and intentional communication in young free-ranging orangutans. Child Development.

[CR6] Bard KA, Dunbar S, Maguire-Herring V, Veira Y, Hayes KG, McDonald K (2014). Gestures and social-emotional communicative development in chimpanzee infants. American Journal of Primatology.

[CR7] Bates E, Camaioni L, Volterra V (1975). The acquisition of performatives prior to speech. Merrill-Palmer Quarterly of Behavior and Development.

[CR8] Beery, S., Morris, D., & Yang, S. (2019). Efficient pipeline for camera trap image review. *arXiv preprint arXiv:1907.06772*

[CR9] Boersma, P., & Weenink, D (2022). Praat: Doing phonetics by computer [Computer program]. Version 6.2.20, retrieved 24 September 2022 from http://www.praat.org/

[CR10] Bourjade M, Cochet H, Molesti S, Guidetti M (2020). Is conceptual diversity an advantage for scientific inquiry? A case study on the concept of ‘gesture’ in comparative psychology. Integrative Psychological and Behavioral Science.

[CR11] Burghardt GM, Bartmess-LeVasseur JN, Browning SA, Morrison KE, Stec CL, Zachau CE, Freeberg TM (2012). Perspectives–minimizing observer bias in behavioral studies: A review and recommendations. Ethology.

[CR12] Cartmill EA, Byrne RW (2007). Orangutans modify their gestural signaling according to their audience's comprehension. Current Biology.

[CR13] Cartmill EA, Byrne RW (2010). Semantics of primate gestures: Intentional meanings of orangutan gestures. Animal Cognition.

[CR14] Dafreville M, Hobaiter C, Guidetti M, Sillam-Dussès D, Bourjade M (2021). Sensitivity to the communicative partner's attentional state: A developmental study on mother–infant dyads in wild chimpanzees (*Pan troglodytes schweinfurthii*). American Journal of Primatology.

[CR15] Eaton, T., Hutton, R., Leete, J., Lieb, J., Robeson, A., & Vonk, J. (2018). Bottoms-up! Rejecting top-down human-centered approaches in comparative psychology. *International Journal of Comparative Psychology, 31*.

[CR16] ELAN (Version 6.4) [Computer software]. (2022). Nijmegen: Max Planck Institute for Psycholinguistics, The Language Archive. Retrieved from https://archive.mpi.nl/tla/elan

[CR17] Farrar, B., Krupenye, C., Motes-Rodrigo, A., Tennie, C., Fischer, J., Altschul, D., & Ostojic, L. (2021). Replication and Reproducibility in Primate Cognition Research

[CR18] Favaro L, Gamba M, Cresta E, Fumagalli E, Bandoli F, Pilenga C, Reby D (2020). Do penguins’ vocal sequences conform to linguistic laws?. Biology letters.

[CR19] Ferrer-i-Cancho R, Bentz C, Seguin C (2022). Optimal coding and the origins of Zipfian laws. Journal of Quantitative Linguistics.

[CR20] Friard O, Gamba M (2016). BORIS: A free, versatile open-source event-logging software for video/audio coding and live observations. Methods in Ecology and Evolution.

[CR21] Fröhlich M, Kuchenbuch P, Müller G, Fruth B, Furuichi T, Wittig RM, Pika S (2016). Unpeeling the layers of language: Bonobos and chimpanzees engage in cooperative turn-taking sequences. Scientific Reports.

[CR22] Fröhlich M, van Schaik CP, van Noordwijk MA, Knief U (2022). Individual variation and plasticity in the infant-directed communication of orang-utan mothers. Proceedings of the Royal Society B.

[CR23] Fröhlich M, Hobaiter C (2018). The development of gestural communication in great apes. Behavioral Ecology and Sociobiology.

[CR24] Genty E (2019). Vocal–gestural combinations in infant bonobos: New insights into signal functional specificity. Animal Cognition.

[CR25] Genty E, Breuer T, Hobaiter C, Byrne RW (2009). Gestural communication of the gorilla (*Gorilla gorilla*): Repertoire, intentionality and possible origins. Animal Cognition.

[CR26] Gilmore RO, Adolph KE (2017). Video can make behavioural science more reproducible. Nature Human Behaviour.

[CR27] Graham KE, Furuichi T, Byrne RW (2017). The gestural repertoire of the wild bonobo (*Pan paniscus*): A mutually understood communication system. Animal Cognition.

[CR28] Graham KE, Furuichi T, Byrne RW (2020). Context, not sequence order, affects the meaning of bonobo (*Pan paniscus*) gestures. Gesture.

[CR29] Graham KE, Badihi G, Safryghin A, Grund C, Hobaiter C (2022). A socio-ecological perspective on the gestural communication of great ape species, individuals, and social units. Ethology Ecology & Evolution.

[CR30] Grund, C., Robbins, M. M., Hobaiter, C. (in preparation) The gestural communication of mountain gorillas.

[CR31] Gupta S, Sinha A (2019). Gestural communication of wild bonnet macaques in the Bandipur National Park. Southern India. Behavioural Processes.

[CR32] Heesen R, Bangerter A, Zuberbühler K, Rossano F, Iglesias K, Guéry JP, Genty E (2020). Bonobos engage in joint commitment. *Science*. Advances.

[CR33] Heesen R, Hobaiter C, Ferrer-i-Cancho R, Semple S (2019). Linguistic laws in chimpanzee gestural communication. Proceedings of the Royal Society B.

[CR34] Hobaiter C, Byrne RW (2011). The gestural repertoire of the wild chimpanzee. Animal Cognition.

[CR35] Hobaiter C, Byrne RW (2011). Serial gesturing by wild chimpanzees: Its nature and function for communication. Animal Cognition.

[CR36] Hobaiter C, Byrne RW (2017). What is a gesture? A meaning-based approach to defining gestural repertoires. Neuroscience & Biobehavioral Reviews.

[CR37] Hobaiter, C., Badihi, G., Daly, Gabriela, B. D. M., Eleuteri, V., Graham, K. E., Grund, C., Henderson, M., Rodrigues, E. D., Safryghin, A., Soldati, A., & Wiltshire, C. (2021). The Great Ape Dictionary video database (1.0.0) [Data set]. *Zenodo*. 10.5281/zenodo.5600472

[CR38] Kaufman AB, Rosenthal R (2009). Can you believe my eyes? The importance of interobserver reliability statistics in observations of animal behaviour. Animal Behaviour.

[CR39] Keen SC, Odom KJ, Webster MS, Kohn GM, Wright TF, Araya-Salas M (2021). A machine learning approach for classifying and quantifying acoustic diversity. Methods in Ecology and Evolution.

[CR40] Kendon, A. (2004). *Gesture: Visible action as utterance*. Cambridge University Press

[CR41] Knox A, Markx J, How E, Azis A, Hobaiter C, van Veen FJ, Morrogh-Bernard H (2019). Gesture use in communication between mothers and offspring in wild orang-utans (*Pongo pygmaeus wurmbii*) from the Sabangau Peat-Swamp Forest, Borneo. International Journal of Primatology.

[CR42] Leavens DA, Hopkins WD (1998). Intentional communication by chimpanzees: A cross-sectional study of the use of referential gestures. Developmental Psychology.

[CR43] Leavens DA, Russell JL, Hopkins WD (2005). Intentionality as measured in the persistence and elaboration of communication by chimpanzees (*Pan troglodytes*). Child development.

[CR44] Liebal K, Call J, Tomasello M (2004). Use of gesture sequences in chimpanzees. American Journal of Primatology: Official Journal of the American Society of Primatologists.

[CR45] Liebal K, Pika S, Tomasello M (2006). Gestural communication of orangutans (*Pongo pygmaeus*). Gesture.

[CR46] Liebal K, Slocombe KE, Waller BM (2022). The language void 10 years on: Multimodal primate communication research is still uncommon. Ethology Ecology & Evolution.

[CR47] Luef EM, Liebal K (2012). Infant-Directed Communication in Lowland Gorillas (*Gorilla gorill*a): Do Older Animals Scaffold Communicative Competence in Infants?. American Journal of Primatology.

[CR48] McCarthy MS, Jensvold MLA, Fouts DH (2013). Use of gesture sequences in captive chimpanzee (*Pan troglodytes*) play. Animal Cognition.

[CR49] Mielke, A., Badihi, G., Graham, K. E., Grund, C., Safryghin, A., Hobaiter, C. (in prep). Many morphs: Establishing great ape gestural repertoires from the bottom-up

[CR50] Molesti S, Meguerditchian A, Bourjade M (2020). Gestural communication in olive baboons (*Papio anubis*): Repertoire and intentionality. Animal Cognition.

[CR51] Noldus LP, Trienes RJ, Hendriksen AH, Jansen H, Jansen RG (2000). The Observer Video-Pro: New software for the collection, management, and presentation of time-structured data from videotapes and digital media files. Behavior Research Methods, Instruments, & Computers.

[CR52] Open Science Collaboration (2015). Estimating the reproducibility of psychological science. Science.

[CR53] Péter, A. (2011). Solomon Coder (version beta 11.01. 22): A simple solution for behavior coding. *Computer programm available at http://solomoncoder. com*

[CR54] Pika S, Liebal K, Tomasello M (2005). Gestural communication in subadult bonobos (*Pan paniscus*): Repertoire and use. American Journal of Primatology: Official Journal of the American Society of Primatologists.

[CR55] Pika S, Liebal K, Tomasello M (2003). Gestural communication in young gorillas (*Gorilla gorilla*): Gestural repertoire, learning, and use. American Journal of Primatology: Official Journal of the American Society of Primatologists.

[CR56] Pika S, Bugnyar T (2011). The use of referential gestures in ravens (*Corvus corax*) in the wild. Nature Communications.

[CR57] Pika S, Fröhlich M (2018). Gestural acquisition in great apes: The social negotiation hypothesis. Animal Cognition.

[CR58] Pollick AS, De Waal FB (2007). Ape gestures and language evolution. Proceedings of the National Academy of Sciences.

[CR59] Revelle W (2022). *psych: Procedures for Psychological, Psychometric, and Personality Research*. Northwestern University, Evanston, Illinois. R package version 2.2.9, https://CRAN.R-project.org/package=psych

[CR60] Roberts AI, Vick SJ, Roberts SGB, Buchanan-Smith HM, Zuberbühler K (2012). A structure-based repertoire of manual gestures in wild chimpanzees: Statistical analyses of a graded communication system. Evolution and Human Behavior.

[CR61] Rodrigues ED, Santos AJ, Veppo F, Pereira J, Hobaiter C (2021). Connecting primate gesture to the evolutionary roots of language: A systematic review. American Journal of Primatology.

[CR62] Rutz C, Webster MM (2021). Ethology adopts the STRANGE framework for animal behaviour research, to improve reporting standards. Ethology.

[CR63] Safryghin A, Cross C, Fallon B, Heesen R, Ferrer-i-Cancho R, Hobaiter C (2022). Variable expression of linguistic laws in ape gesture: A case study from chimpanzee sexual solicitation. Royal Society Open Science.

[CR64] Schofield D, Nagrani A, Zisserman A, Hayashi M, Matsuzawa T, Biro D, Carvalho S (2019). Chimpanzee face recognition from videos in the wild using deep learning. Science Advances.

[CR65] Scott NM, Pika S (2012). A call for conformity: Gesture studies in human and non-human primates. Developments in Primate Gesture Research.

[CR66] Seyfarth R, Cheney D (2018). Pragmatic flexibility in primate vocal production. Current Opinion in Behavioral Sciences.

[CR67] Schel, A. M., Bono, A., Aychet, J., Pika, S., & Lemasson, A. (2022). Intentional gestural communication amongst red-capped mangabeys (*Cercocebus torquatus*). *Animal Cognition*, 1–18. 10.1007/s10071-022-01615-7 PMC9617956 35362785

[CR68] Schweinfurth MK, Call J (2021). Capuchins (*Sapajus apella*) and their Aversion to Inequity. *Comparative Cognition*.

[CR69] Slocombe KE, Waller BM, Liebal K (2011). The language void: The need for multimodality in primate communication research. Animal Behaviour.

[CR70] Tomasello M, George BL, Kruger AC, Jeffrey M, Evans A (1985). The development of gestural communication in young chimpanzees. Journal of Human Evolution.

[CR71] Townsend SW, Koski SE, Byrne RW, Slocombe KE, Bickel B, Boeckle M, Manser MB (2017). Exorcising Grice's ghost: An empirical approach to studying intentional communication in animals. Biological Reviews.

[CR72] Vail AL, Manica A, Bshary R (2013). Referential gestures in fish collaborative hunting. Nature Communications.

[CR73] Wadewitz P, Hammerschmidt K, Battaglia D, Witt A, Wolf F, Fischer J (2015). Characterizing vocal repertoires—Hard vs. soft classification approaches. PloS One.

[CR74] Webster, M. M., & Rutz, C. (2020). How STRANGE are your study animals? *Nature*, 337–340. 10.1038/d41586-020-01751-5 32541916

[CR75] Wiltshire, C., Lewis-Cheetham, J., Komedová, V., Matsuzawa, T., Graham, K. E., & Hobaiter, C. (in review). DeepWild: Application of the pose estimation tool DeepLabCut for behaviour tracking in wild chimpanzees and bonobos. *Journal of Animal Ecology* 10.1111/1365-2656.13932 37165474

[CR76] Zipf, G. K. (1949). Human behavior and the principle of least effort: An introduction to human ecology. Cambridge

